# Exploring the genetic and socioeconomic interplay between ADHD and anxiety disorders using Mendelian randomization

**DOI:** 10.3389/fpsyt.2024.1439474

**Published:** 2024-08-06

**Authors:** Xiaojuan Deng, Hongyan Ren, Shuang Wu, Huijin Jie, Chengyu Gu

**Affiliations:** ^1^ Mental Health Center of West China Hospital, Sichuan University, Chengdu, Sichuan, China; ^2^ Department of Psychiatry, The Fourth People’s Hospital Of Haining, Haining, Zhejiang, China

**Keywords:** ADHD, anxiety disorders, Mendelian randomization, income, educational attainment, intelligence

## Abstract

**Background:**

ADHD and anxiety disorders often co-occur, sharing symptoms and dysfunctions, yet the underlying mechanisms remain elusive.

**Methods:**

To explore the shared and distinct genetic variations between ADHD and anxiety disorders, we applied Mendelian randomization (MR) analysis to ADHD, anxiety disorders, and three socioeconomic factors: income, educational attainment (EA), and intelligence. MR analysis utilized genome-wide association study summary datasets (anxiety disorder: 7,016 cases and 14,745 controls; ADHD: 38,691 cases and 275,986 controls; EA: 766,345 participants; intelligence: 146,808 participants; household income: 392,422 participants), with inverse-variance weighting as the primary method.

**Results:**

Our MR analysis revealed no discernible genetic-level causal effect between ADHD and anxiety disorders (p > 0.77). Additionally, the independent variables for ADHD (25 SNPs) and anxiety disorders (18 SNPs) did not overlap, highlighting the genetic distinction between the two conditions. Higher income (p < 0.002) and EA (p < 0.005) were found to serve as protective factors for both ADHD and anxiety disorders. Genetic predisposition to higher income (86 SNPs) and EA (457 SNPs) were identified as a potential common protective factors for both conditions. Lastly, genetic predisposition to higher intelligence was found to potentially guard against ADHD (p < 0.001) but not against anxiety disorders (p > 0.55).

**Conclusion:**

Our findings indicate that the shared symptoms observed between ADHD and anxiety disorders are more likely influenced by genetic predispositions related to socioeconomic factors rather than by the genetic predispositions specific to the disorders themselves.

## 1 Introduction

ADHD, or Attention Deficit Hyperactivity Disorder, is a common neurodevelopmental disorder that affects both children and adults, with a global prevalence of approximately 5% in children and 2.5% in adults (1). ADHD can have profound effects on academic, social, and occupational outcomes, highlighting the importance of early diagnosis and intervention for long-term prognosis and quality of life (2). Personalized treatment strategies combining medication, therapy, and behavioral interventions are essential for managing ADHD symptoms and enhancing quality of life (3, 4). The development of ADHD is influenced by a complex interplay of genetic predisposition, environmental exposures such as prenatal tobacco exposure and child maltreatment, and neurobiological abnormalities (5).

Anxiety disorders are highly prevalent mental health conditions globally, impacting an estimated 264 million individuals, with varying rates among different demographics and a higher incidence in women (6, 7). In the United States, approximately 31% of adults will experience an anxiety disorder at some stage in their lives (8). The burden of anxiety disorders on public health systems and society is substantial, manifested through increased healthcare utilization, reduced quality of life, and the presence of comorbidities (9). Effective treatment strategies, such as cognitive-behavioral therapy and medication, are crucial for enhancing overall well-being (10). Genetic predisposition, environmental stressors, and brain chemistry imbalances contribute to anxiety disorders development, with risk factors including family history, trauma, chronic stress, and certain medical conditions (11). Tailored interventions, like the ‘screen-and-treat’ approach, and ongoing research efforts are essential for addressing the complexities of anxiety disorders (12–14).

Although ADHD and anxiety disorders distinct each other from their classification, diagnostic properties, and treatment (1), these two diseases often co-occur with each other, worsening symptoms and function. Shared developmental origins may complicate diagnosis and treatment, affecting medication use and cardiometabolic risk, especially in autistic individuals (1, 15, 16). There has been frequent misdiagnosis of anxiety disorders and ADHD before an autism diagnosis, particularly in women. This underscores the complex relationship and diagnostic challenges between these conditions and the need for improved practitioner awareness (17).

Moreover, ADHD increases the risk of anxiety disorders, with symptoms overlapping and exacerbating each other, leading to impaired daily functioning. Coexistence of ADHD and anxiety affects emotional well-being, possibly influenced by genetic variants (3, 18). However, whether causal-relationship exists or not remain uncertain.

We hypothesize that both shared and distinct genetic variations exist between ADHD and anxiety disorders, which determine their shared and unique clinical features. To disentangle these relationships, we conducted a bidirectional Mendelian randomization (MR) analysis between ADHD and anxiety disorders to directly test the effect of genetic liability to one disease on the other. Additionally, we employed MR analysis to examine the impact of common socioeconomic-related factors, including income, educational attainment (EA), and intelligence, on both ADHD and anxiety disorders. Our study aims to enhance the understanding of the interplay and distinctions between ADHD and anxiety disorders.

## 2 Methods

The workflow was carried out as follows: First, a two-sample MR analysis was performed to investigate the causal relationship between ADHD and anxiety disorders, using independent instrumental variables (IVs) for the study. Next, a functional annotation analysis was undertaken to examine the roles of the selected IVs and corresponding genes in relation to ADHD and anxiety disorders. Additionally, an MR analysis was conducted to assess the impact of three socioeconomic factors-educational attainment (EA), income, and intelligence-on ADHD and anxiety disorders. This was done to understand how these common factors influence both conditions.

### 2.1 GWAS summary data for MR analysis

The anxiety disorder and ADHD summary GWAS datasets were sourced from the Psychiatric Genomics Consortium (PGC). The anxiety disorder data comprise 7,016 cases and 14,745 controls (19), with samples come from USA, Switzerland, Netherlands, Germany, and Australia. The ADHD dataset comprises 38,691 cases and 275,986 controls, all of European ancestry (20). The datasets on educational attainment (EA) included 766,345 participants (21), with EA measured by the number of years of schooling completed. All association analyses were conducted at the cohort level in samples limited to individuals of European descent. Additionally, GWAS datasets for intelligence (fluid intelligence score) and household income were obtained from Yang Lab (https://yanglab.westlake.edu.cn/) (22), involving 146,808 and 392,422 participants, respectively. All the participants in the datasets were of European origin. Please note that education year, income level, and intelligence level are quantitative traits. Unlike case/control studies, GWAS datasets with quantitative traits were analyzed using linear regression to identify significant SNPs as IVs.

### 2.2 MR analysis

The primary MR analysis was conducted using the inverse-variance weighted (IVW) method as main method, with additional support from the weighted median (WM) and MR-Egger methods provided by TwoSampleMR (23). Single-nucleotide polymorphisms (SNPs) linked to outcome (P < 5×10^–5^) were chosen from exposure as potential genetic variants.

To ensure the reliability of instrumental variables (IVs), we meticulously selected SNPs from GWAS datasets based on their genome-wide significance (p < 5.00E-08). When genome-wide significance was not met or the number of suitable instruments was 10 or fewer, we carefully adjusted the threshold for selecting IVs in the MR approach to a p-value of 1.00E-05. This threshold was chosen because a more relaxed threshold would result in IVs with weaker effects, potentially compromising the reliability of MR analysis results (23). These selected SNPs were further refined within a 10 Mb window using a clustering r2 cutoff of 0.001. In each MR analysis, we systematically excluded SNPs absent in the outcome dataset, those with intermediate allele frequencies, and redundant SNPs. This stringent curation process ensured the quality and reliability essential for the MR analysis.

In addition, the intercept from the MR-Egger model was used to assess directional pleiotropy. Heterogeneity was assessed using Cochran’s Q test and I^2^ statistics, with significance thresholds set at P < 0.05 and I^2^ > 0.25 (24).

### 2.3 Annotation analysis

To understand the genetic instrumental variables (SNPs) chosen for the MR analysis regarding ADHD and anxiety disorders, we initially mapped these SNPs to genes utilizing the SNP-Gene mapping tools provided by AIC LLC (https://www.gousinfo.com/en/snpmap2gene.html). Then, we performed annotation analysis using the ‘Functional Annotation Tool’ of DAVID (https://david.ncifcrf.gov) and literature data mining (LDM) tools from AIC LLC (https://www.gousinfo.com/en/advancedsearch.html). These tools were employed to scrutinize the genetic variants and their associated genes. The functional analysis primarily concentrated on examining the individual functions of these genes or genetic variants and their connections to anxiety disorders and ADHD. In DAVID, we utilized three gene ontologies (GOTERM_BP_DIRECT, GOTERM_CC_DIRECT, and GOTERM_MF_DIRECT) and three pathways (REACTOME_PATHWAY, WIKIPATHWAYS, and KEGG_PATHWAY). The pathways or functional groups that these genes are enriched in will help in understanding the function of the corresponding genes. Concurrently, the AIC LDM tools were used to investigate existing scientific literature linking these genetic variants and genes to ADHD and anxiety disorders. Specifically, LDM was conducted for each relationship between the diseases, genes, and SNPs with the purpose of identifying supporting scientific references from a wide range of sources, including scientific literature (PubMed, arXiv, and bioRxiv), scientific databases (GEO, GenBank, Protein Data Bank (PDB), and Ensembl), and documents and reports from research organizations (the World Health Organization (WHO), National Institutes of Health (NIH), and Centers for Disease Control and Prevention (CDC)) (https://www.gousinfo.com/en/userguide.html).

## 3 Results

### 3.1 MR analysis for ADHD and anxiety disorders

We found that genetic liability to anxiety disorder was not associated with the risk of ADHD (p > 0.83). Meanwhile, genetic liability to ADHD was not associated with the risk of anxiety disorder (p > 0.55). As shown in **Table 1**, 25IVs were selected for ADHD, and 18 for anxiety disorders. However, none of the three methods (IVW, WM, and MR-Egger) showed statistical significance. These results indicate that while the selected IVs are related to ADHD or anxiety disorders, they do not influence the other condition.

**Table 1:** Mendelian randomization analysis results for ADHD and AD.

Exposure	Outcome	Method	N_IV	P_IV	b (se)	OR [95%CI]	P
ADHD	Anxiety	IVW	25	5.00E-08	0.014 (0.046)	1.01 [0.93-1.11]	0.77
		WM	25	5.00E-08	-0.039 (0.065)	0.96 [0.85-1.09]	0.55
		MR-Egger	25	5.00E-08	0.048 (0.158)	1.05 [0.77-1.43]	0.77
Anxiety	ADHD	IVW	18	1.00E-05	0.001 (0.018)	1.00 [0.97-1.04]	0.95
		WM	18	1.00E-05	0.005 (0.024)	1.01 [0.96-1.05]	0.83
		MR-Egger	18	1.00E-05	0.005 (0.026)	1.00 [0.95-1.06]	0.87

**Figure 1:**
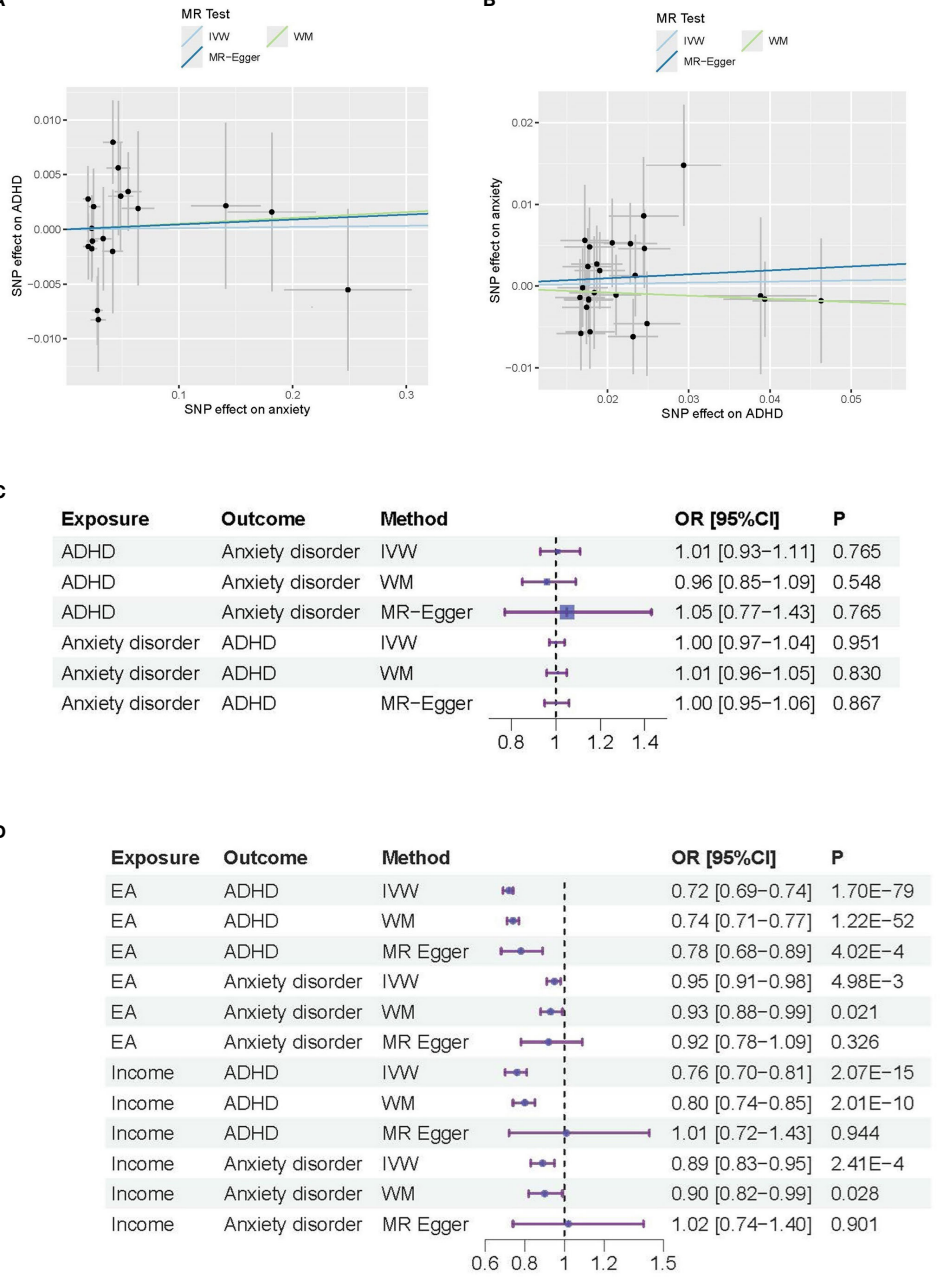
MR analysis results for ADHD and anxiety disorders. **(A)** Causal effects of anxiety disorder on ADHD; **(B)** Causal effects of ADHD on anxiety disorder. **(C)** Forest plot of the causal effects between ADAD and anxiety disorders. **(D)** Forest plot of the causal effects of Educational attendance (EA) and Income on ADAD and anxiety disorders.

The heterogeneity analysis suggests that the directions of causal effects across the set of applied techniques were largely the same. No directional pleiotropy (P > 0.05 and MR-Egger intercept < 0.01) or heterogeneity (P > 0.05) was detected. We provided the scatter plot and the forest plot of the bidirectional MR analysis in **Figure 1**.

In **Figure 1A**, the Scatter plot depicts the associations between genetic variants and anxiety disorders (X-axis) and ADHD (Y-axis) used in the MR analysis. Each point represents an individual genetic variant (SNP). The slope of the regression line (solid line) indicates the estimated causal effect of anxiety on ADHD. The R-squared value is about 17%, indicating the proportion of variance in ADHD explained by the genetic variants via anxiety disorders. The intercept of the regression line is about 0, representing the average pleiotropic effect across SNPs.

In **Figure 1B**, the Scatter plot depicts the associations between genetic variants and ADHD (X-axis) and anxiety disorders (Y-axis) used in the MR analysis, with R-squared value of about 17% and regression line intercept of about 0.

**Figures 1C**, **D** are the Forest plot of the effect sizes (odds ratios) for the association between different exposure and outcome. The horizontal lines represent the 95% confidence intervals (CIs) for each study. The size of the square for each study represents the weight of the study in the meta-analysis. P-values indicate the statistical significance of the effect sizes. In **Figure 1C**, the p values are not significant (p-value>0.05), which indicate that the effect observed in the study could be due to random variation or chance. In **Figure 1D**, the p values are significant for the main MR methods (IVW method; p-value<0.005), which indicate that both Educational Attainment (EA) and Income serve as common influential factors for both ADHD and anxiety disorders.

### 3.2 Annotation for IVs and corresponding genes

**Table 2** lists the IVs and their mapped genes for ADHD and anxiety disorders. For ADHD, 25 SNPs are associated with genes such as ANO10, SORCS3, CDH8, TEX41, FOXP1, COL19A1, and others. Certain SNPs (e.g., rs1162202 and rs115111850) are found to be associated with multiple genes. For instance, rs1162202 is linked to genes LOC105371305 and CDH8. For anxiety disorders, 18 SNPs are linked to genes including CAMKMT, ARPP19, SOCS5, TNS3, VWDE, TSHZ2, and others. Some SNPs like rs62516012 are mapped to multiple genes, such as CASC21 and CASC8. Additionally, a few SNPs are not associated with any gene. Importantly, the SNPs for ADHD and anxiety disorders presented zero overlap, indicating distinct genetic markers for each condition. This table highlights the genetic distinctions between ADHD and anxiety disorders through their respective IVs and gene mappings.

**Table 2:** List of IVs and mapped genes for ADHD and anxiety disorders.

IVs of ADHD		IVs of Anxiety disorders	
SNP Name	Gene Symbol	SNP Name	Gene Symbol
rs115111850	ANO10	rs1067394	CAMKMT
rs11596214	SORCS3	rs116274579	ARPP19
rs1162202	LOC105371305	rs11998109	LOC105377795
rs1162202	CDH8	rs13340324	LINC02263
rs1438898	TEX41	rs17823065	SOCS5
rs17718444	FOXP1	rs4724582	TNS3
rs2025286	COL19A1	rs56242606	VWDE
rs2582895	LINC02758	rs6068466	TSHZ2
rs4916723	MIR9-2HG	rs62156215	LMAN2L
rs4925811	ARHGAP39	rs62516012	CASC21
rs549845	PTPRF	rs62516012	CASC8
rs6537401	LSM6	rs72850179	NCKAP5
rs73145587	LOC105375341	rs79310980	LMCD1-AS1
rs7506904	DCC	rs9949003	ATP9B
rs76284431	LOC105370656	rs11190870	/
rs9969232	FOXP2	rs112311059	/
rs10875612	/	rs113789029	/
rs11255890	/	rs759707	/
rs17576773	/	rs7910612	/
rs2886697	/	/	/
rs6082363	/	/	/
rs704061	/	/	/
rs7613360	/	/	/
rs76857496	/	/	/
rs77960	/	/	/
rs7844069	/	/	/

**Figure 2:**
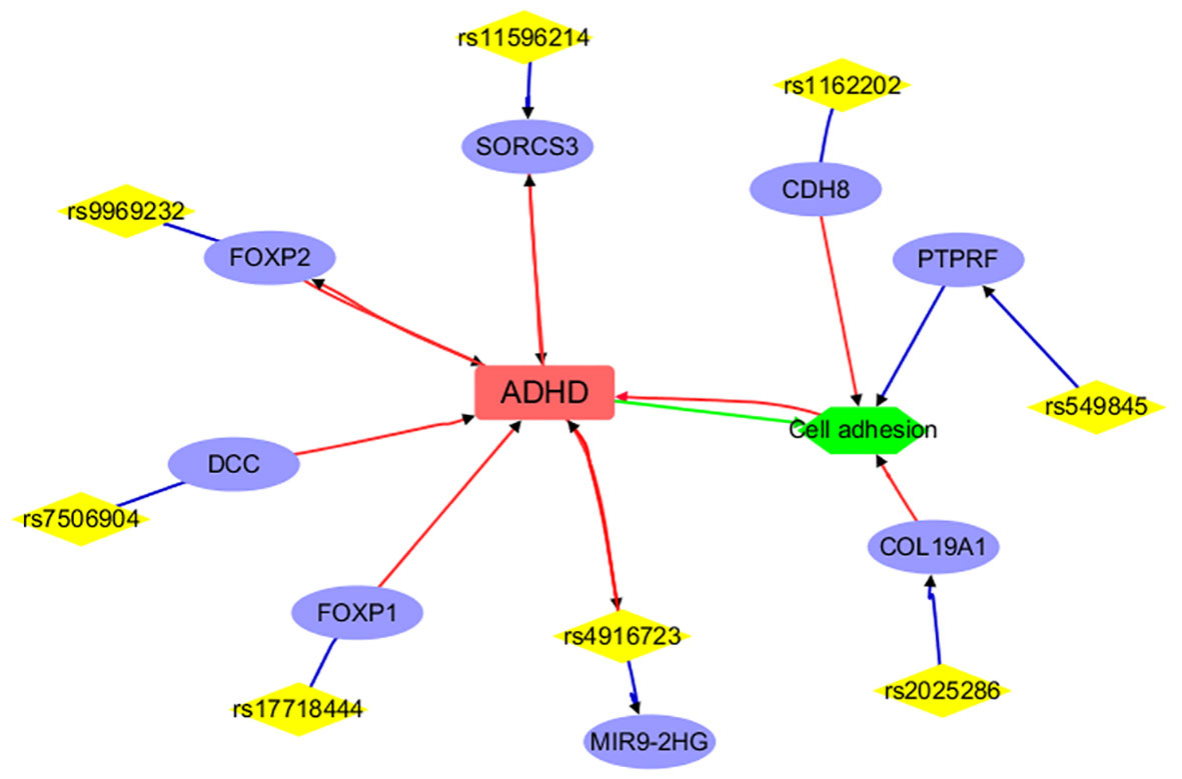
Annotation results for the IVs selected and mapped genes from ADHD. Edges in red and green represent positive and negative relationships, respectively. Edges in blue represent no polarity.

**Figure 3:**
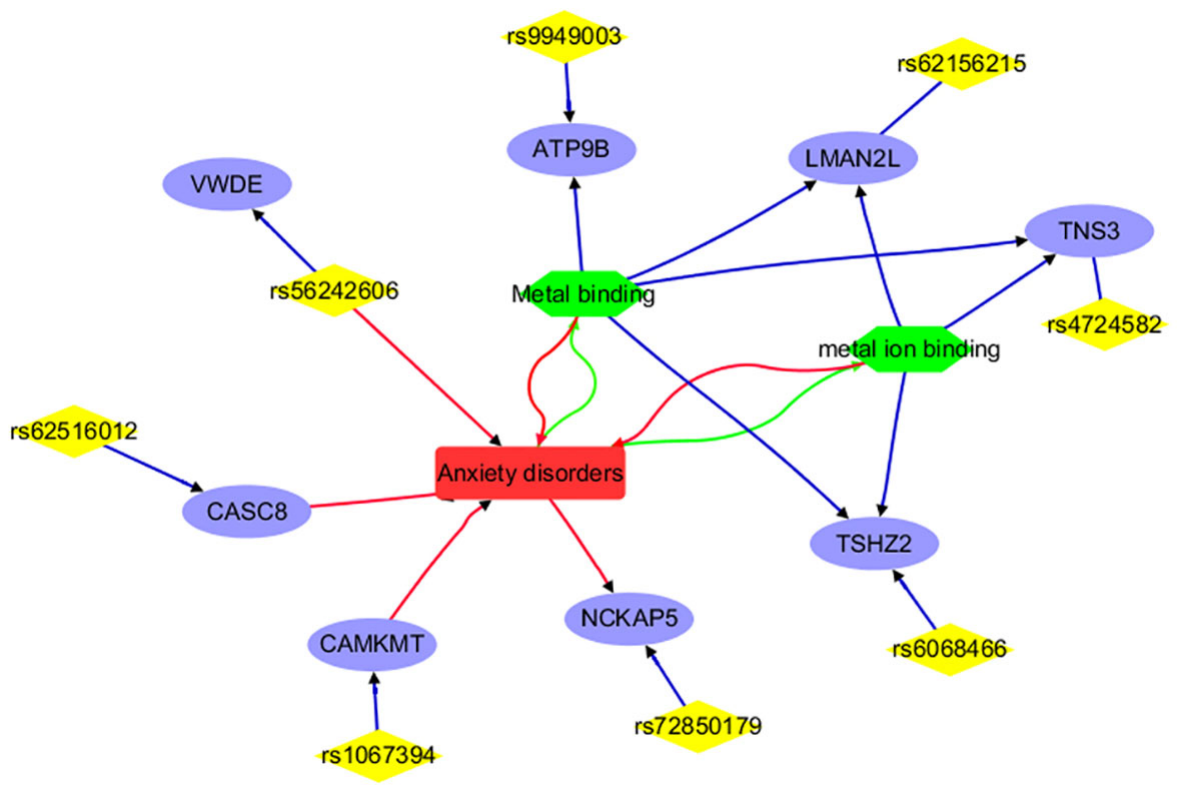
Annotation results for the IVs selected and mapped genes from Anxiety disorders. Edges in red and green represent positive and negative relationships, respectively. Edges in blue represent no polarity.

To understand the role of the IVs and mapped genes in ADHD and AD, we first used DAVID to annotate the function of the mapped genes at an individual level, identifying the pathways and cellular processes these genes are involved in. Then, we used LDM tools from AIC LLC (https://www.gousinfo.com/en/advancedsearch.html) to explore the connections of these SNPs, genes, and cellular processes to ADHD and AD. Based on these results, we constructed two networks centered on the two conditions, as shown in **Figures 2**, **3**, respectively.

**Figure 2** presents a detailed network illustrating the intricate connections among ADHD, specific genetic variations (SNPs), mapped genes, and cellular processes. Direct associations with ADHD are highlighted for genes such as FOXP2, SORCS3, FOXP1, DCC, CDH8, and a specific SNP (rs4916723), along with the cellular process of ‘Cell adhesion.’ Conversely, genes like ANO10, TEX41, and LOC105371305 indirectly implicate ADHD through their genetic variations. Moreover, genes like PTPRF, CDH8, and COL19A1, known to participate in cell adhesion, potentially establish connections to ADHD. This comprehensive network effectively delineates the genetic and cellular landscape related to ADHD but not anxiety disorders and showcases both direct and indirect associations. Please note that only 8 out of the 25 IVs (SNPs) used in MR analysis have been included in the network, suggesting that more research is needed to study the remaining IVs and their connections with ADHD.

To note, each edge depicted in **Figures 2**, **3** signifies a relationship between the two entities it links. In the case of connections between diseases (ADHD or anxiety disorder), cell processes (such as cell adhesion, metal binding, and metal ion binding), and genes, the edge represents an association supported by existing literature. When it comes to genes and SNPs, it indicates that the SNP has been mapped to that specific gene.

**Figure 3** portrays a network illustrating the interconnections between anxiety disorders and its associated IVs (SNPs), mapped genes, and biological processes. Notably, entities such as CAMKMT, NCKAP5, CASC8, and rs56242606 exhibit positive associations with anxiety disorders. Conversely, anxiety disorders demonstrate negative relationships with the biological processes of Metal binding and metal ion binding. This comprehensive network offers insights into the genetic and cellular landscape specific to anxiety disorders, distinguishing it from ADHD, and showcasing both direct and indirect associations. It’s important to highlight that only 8 out of the 18 IVs (SNPs) utilized in MR analysis are represented in the network, underscoring the need for further research to explore the remaining IVs and their connections with anxiety disorders.

### 3.3 Influence of socioeconomic related factors on AD and ADHD

To explore the mechanism of the common influential factors on AD and ADHD, we also conducted a one-way MR analysis to study the effect of three important factors, namely educational attainment (EA), intelligence and income, on both ADHD and AD. The MR analysis has been done in a previous study (25) and we were able to replicate the process in this study. We present the details of the results in **Table 3** and expand the discussion based on it.

**Table 3:** Causal effects of EA, Income, and Intelligence on ADHD and AD.

Exposure	Outcome	Method	N_IV	P_IV	b (se)	OR [95%CI]	P
EA	ADHD	IVW	457	5E-8	-0.333 (0.018)	0.72 [0.69-0.74]	<0.001
		WM	457	5E-8	-0.300 (0.020)	0.74 [0.71-0.77]	<0.001
		MR Egger	457	5E-8	-0.249 (0.070)	0.78 [0.68-0.89]	<0.001
EA	AD	IVW	458	5E-8	-0.056 (0.020)	0.95 [0.91-0.98]	0.005
		WM	458	5E-8	-0.070 (0.030)	0.93 [0.88-0.99]	0.021
		MR Egger	458	5E-8	-0.084 (0.085)	0.92 [0.78-1.09]	0.326
Income	ADHD	IVW	86	5E-8	-0.280 (0.035)	0.76 [0.70-0.81]	<0.001
		WM	86	5E-8	-0.229 (0.036)	0.80 [0.74-0.85]	<0.001
		MR Egger	86	5E-8	0.012 (0.175)	1.01 [0.72-1.43]	0.944
Income	AD	IVW	87	5E-8	-0.121 (0.033)	0.89 [0.83-0.95]	<0.001
		WM	87	5E-8	-0.103 (0.047)	0.90 [0.82-0.99]	0.028
		MR Egger	87	5E-8	0.020 (0.162)	1.02 [0.74-1.40]	0.901
Intelligence	ADHD	IVW	78	5E-8	-0.158 (0.028)	0.85 [0.81-0.90]	<0.001
		WM	78	5E-8	-0.113 (0.026)	0.89 [0.85-0.94]	<0.001
		MR Egger	78	5E-8	-0.060 (0.124)	0.94 [0.74-1.20]	0.632
Intelligence	AD	IVW	76	5E-8	-0.005 (0.026)	0.99 [0.95-1.05]	0.833
		WM	76	5E-8	-0.021 (0.036)	0.98 [0.91-1.05]	0.555
		MR-Egger	76	5E-8	-0.153 (0.148)	0.86 [0.64-1.15]	0.306

In MR-Egger analysis, the Egger intercept values range from -0.005 to 0.004 (see **Supplementary Table 1**). None of the p-values for pleiotropy are below the typical significance threshold of 0.05, indicating there is no evidence of directional pleiotropy in the dataset.

For ADHD, EA, Income, and Intelligence show a consistent protective causal effect across IVW and WM methods (OR ∈ [0.72, 0.95]; p < 0.028). Although MR Egger indicates non-significant pleiotropy (p-values > 0.09), significant heterogeneity is observed in several analyses where Q_p <0.001 and I² >0.5, indicating that the IVs from Income, Intelligence, and EA may not all be valid in estimating the same underlying causal effect on ADHD.

For anxiety disorders, EA and Income show a consistent protective causal effect across IVW and WM methods (OR ∈ [0.89, 0.95]; p < 0.033), while Intelligence shows no significant effect (OR ∈ [0.86, 0.99]; p > 0.555). MR Egger results indicate no significant pleiotropy (p-values > 0.091). Additionally, heterogeneity is generally low across analyses, as indicated by Q_p >0.4 and I²<0.01, suggesting that the IVs from Income, Intelligence, and EA are likely valid in estimating the causal effect on anxiety disorders.

The MR analysis results indicate that both Educational Attainment (EA) and Income serve as common influential factors for both ADHD and anxiety disorders, as shown in **Figure 1D**. Notably, while a large number of instruments from both Income (>86 IVs) and EA (>458 IVs) may not all accurately estimate the same underlying causal effect on ADHD, all IVs appear valid in estimating the underlying causal effect on anxiety disorders. Consequently, we can infer that genetic predispositions to EA and Income may largely serve as common protective factors for both anxiety disorders and ADHD.

In contrast, while Intelligence demonstrates a potential to reduce the risk of ADHD, as indicated by its significant protective effects across various analytical methods, its impact on anxiety disorders appears less clear. The data suggests that Intelligence may not consistently serve as a protective factor for anxiety disorders, as evidenced by non-significant findings across multiple analyses. Thus, genetic predisposition to Intelligence may not uniformly confer protection against both anxiety disorders and ADHD.

## 4 Discussion

While ADHD and anxiety disorders are distinct in their diagnostic criteria and treatment approaches (1), they frequently co-occur, exacerbating symptoms and often leading to misdiagnosis (17). Understanding the shared and unique causal factors and underlying mechanisms of both conditions is crucial for improving diagnosis and treatment outcomes. This study examined the genetic-level causal effects between ADHD and anxiety disorders, as well as their associations with educational attainment (EA), income, and intelligence. The findings revealed both distinct and shared genetic variables influencing both ADHD and anxiety disorders.

Our study found no causal association between anxiety disorder and ADHD at the genetic level, aligning with a recent MR study that also utilized European-origin datasets with a relatively smaller sample size (26). Despite selecting instrumental variables for both conditions, none of the methods used (IVW, WM, and MR-Egger) showed statistical significance. These results suggest that while the selected genetic variables may be related to either ADHD or anxiety, they do not impact the risk of the other condition.

Functional annotation analysis showed that a large part of the IVs and corresponding genes selected for ADHD were already implicated in the disorder (**Figure 2**). Specifically, variations in the FOXP2 gene (27, 28) impact language and cognitive functions, potentially contributing to ADHD traits. The SORCS3 gene (20), FOXP1 gene (29), and the rs4916723 polymorphism (29) are associated with increased ADHD risk, with SORCS3 potentially involved in neurodevelopmental pathways. Additionally, cell adhesion, which is vital for neuronal development, synaptic connectivity, and brain circuitry, may impact ADHD pathophysiology via disruptions in cell adhesion molecules and genetic abnormalities in ganglioside metabolism (30). Cell adhesion-related genes like COL19A1, PTPRF, and CDH8 (31, 32) were implicated in neuronal development and synaptic function and may contribute to ADHD susceptibility through disruptions in cell adhesion molecules and synaptic connectivity. Moreover, it is noted that 17 out of the 25 ADHD IVs (SNPs) used in the MR analysis did not yield results from functional annotation analysis. This indicates the need for further research to investigate the remaining IVs and their connections with ADHD.

For anxiety disorders, 8 out of the 18 IVs (SNPs) or their corresponding genes are highlighted by the functional annotation analysis (**Figure 3**). These include CAMKMT, NCKAP5, CASC8, rs56242606, ATP9B, LMAN2L, TNS3, and TSHZ2. Specifically, CAMKMT influences anxiety disorders through CAMKII methylation, which impacts neuronal function and synaptic plasticity, showing significant genetic associations in European populations (19, 33). NCKAP5 may contribute to the pathophysiology of anxiety disorders by affecting synaptic plasticity and neuronal development (34). CASC8’s role in anxiety disorders is linked to its influence on neural development and function, with the haplotypic block rs4733767 indicating genetic susceptibility (35). The rs56242606 variant (on the VWDE gene) is associated with an increased risk of anxiety disorders, potentially impacting gene regulation related to anxiety and brain traits such as smaller amygdala volume (36).

The relationship between anxiety disorders and metal ion binding is notable; anxiety can disrupt metal homeostasis, leading to oxidative stress, while dysregulated metal binding can exacerbate anxiety through neuronal dysfunction (37, 38). Metal binding affects anxiety disorders by influencing neurotransmitter function and oxidative stress. Interventions such as berberine, bisdemethoxycurcumin (BDMC), and maternal zinc supplementation show promise in alleviating anxiety by modulating metal binding (39–41). Genes related to metal binding or metal ion binding (e.g., ATP9B, LMAN2L, TNS3, and TSHZ2) may play a role in the development of anxiety disorders. These findings suggest the need for further investigation into the remaining 10 IVs to understand their connection with anxiety disorders.

Notably, none of the selected IVs or their mapped genes overlap between ADHD and anxiety disorders. The distinct IVs identified for ADHD and anxiety disorders may illustrate the differences between these two conditions, potentially explaining their distinct diagnostic and treatment approaches.

Our MR analysis unveils Educational Attainment (EA) as a shared influential factor impacting both ADHD and anxiety disorders. Notably, a multitude of instruments from EA (>458 IVs) and Income (>86 IVs) highlight their complex interplay in shaping the risk landscape for these conditions. While the accuracy of estimating the causal effect on ADHD varies across these instruments, all IVs demonstrate validity in estimating the underlying causal effect on anxiety disorders. This suggests a nuanced relationship wherein genetic predisposition to EA and Income may partially converge as common protective factors for both anxiety disorders and ADHD. These findings underscore the intricate role of EA in the etiology of both conditions, prompting further investigation into the mechanisms underlying this association.

While some studies suggested potential relationships between ADHD and Intelligence, the results are non-consistent (42, 43). Our MR analysis results indicate that genetic liability to higher intelligence may protect again ADHD, but not Anxiety disorder.

EA emerges as a pivotal factor influencing both ADHD and anxiety disorders. In the context of ADHD, lower EA is associated with negative academic outcomes, including reduced graduation rates and academic achievement, possibly due to challenges in focus and organization (44). Conversely, for anxiety disorders, higher EA serves as a protective factor, correlated with lower rates of anxiety disorders and offering effective coping mechanisms. In contrast, lower EA correlates with elevated anxiety levels, potentially impeding academic performance (7, 45). Moreover, the interplay between genetic factors, income, and educational attainment further modulates the relationship between ADHD and EA (46, 47). These findings highlight the intricate relationship between EA and both ADHD and anxiety disorders, emphasizing the need for targeted interventions to address academic challenges and mental health concerns in educational settings.

Income serves as another common influential factor for both ADHD and anxiety disorders. In the context of ADHD, lower income levels are associated with a higher prevalence of ADHD diagnosis, potentially influenced by socioeconomic disparities and limited access to healthcare and education (48–50). Additionally, higher family income has been correlated with reduced ADHD symptoms in early childhood, possibly mediated through factors such as asthma and physical fitness. For anxiety disorders, reduced income levels can exacerbate the condition by decreasing productivity and increasing the risk of job loss, ultimately contributing to financial instability (51). Notably, income disparities are particularly evident among vulnerable populations such as migrants and perinatal groups affected by anxiety disorders (52).

The MR analysis of EA and income on ADHD and anxiety disorder not only provides genetic support for the previously observed effects of socioeconomic factors on these disorders but also uncovers potentially shared genetic factors and their influential paths. These findings may offer insights into understanding the shared characteristics and co-occurrence of ADHD and anxiety disorder.

It should be noted that the results of MR analysis vary across different methods. This is expected, as the IVW method generally has the highest statistical power among the three methods, often resulting in significant outcomes. In contrast, the WM and MR-Egger methods have less power, making it more difficult to achieve statistical significance with the same set of IVs and effect sizes (23). Although some datasets showed significant heterogeneity, indicating variability in the causal effects among IVs, no significant direct pleiotropy was detected. This suggests that the IV influences the outcome solely through its effect on the exposure, supporting the validity of our MR analysis.

A key strength of this study is our use of MR analysis to evaluate the causal relationship between ADHD and anxiety disorder at genetic level, as well as potential influencing factors such as educational attainment, income, and intelligence. This approach takes advantage of the random allocation of genetic variants during inheritance, which naturally protects these variables from confounding factors like environmental influences and lifestyle choices. Consequently, MR analysis can provide more robust genetic evidence and strengthen the persuasiveness of causal inferences.

However, this study has several limitations that should be addressed in future research. First, the participants in the datasets used for MR analysis are all of European origin. It is essential to examine data from other racial groups to corroborate the findings of this study. Additionally, the results only reflect the connection and distinction between ADHD and anxiety disorder at the genetic level. Since these two disorders are complex in their pathology, their interplay at different levels (e.g., cellular processes, organ/tissue interactions) should also be studied.

## 5 Conclusion

Our findings suggest that the shared symptoms between ADHD and anxiety disorders are more likely influenced by genetic predispositions related to socioeconomic factors, rather than by genetic predispositions specific to the disorders themselves.

## Data availability statement

The original contributions presented in the study are included in the article. Further inquiries can be directed to the corresponding author.

## Ethics statement

The data utilized in this study are publicly available. The original studies were conducted in accordance with the local legislation and institutional requirements.

## Author contributions

XD: Writing – review & editing, Writing – original draft, Visualization, Resources, Project administration, Methodology, Investigation, Formal analysis, Data curation, Conceptualization. HR: Writing – review & editing, Software, Methodology, Investigation, Formal analysis, Data curation, Conceptualization. SW: Writing – review & editing, Software, Methodology, Investigation, Formal analysis, Data curation, Conceptualization. HJ: Writing – review & editing, Software, Methodology, Investigation, Formal analysis, Data curation, Conceptualization. CG: Conceptualization, Writing – review & editing.
