# Supplementary material for: Exploring the genetic and socioeconomic interplay between ADHD and anxiety disorders using Mendelian randomization
Source: Front Psychiatry. 2024 Aug 6;15:1439474. doi: 10.3389/fpsyt.2024.1439474 (PMC11333326; doi:10.3389/fpsyt.2024.1439474)
Supplement: Supplementary file 1 [file Table_1.docx]

**Supplementary Table 1 The full results of MR analysis for Income, Intelligence and EA on ADHD and anxiety disorders**

| Exposure | Outcome | Method | b (se) | OR [95%CI] | N_IV | Q_P | I2 | Egger_intercept | P_pleiotropy | p |
| --- | --- | --- | --- | --- | --- | --- | --- | --- | --- | --- |
| Intelligence | ADHD | MR-Egger | -0.060 (0.124) | 0.94 [0.74-1.20] | 78 | <0.001 | 0.677 | -0.002 | 0.422 | 0.632 |
| Intelligence | ADHD | IVW | -0.158 (0.028) | 0.85 [0.81-0.90] | 78 | <0.001 | 0.68 | NA |  | <0.001 |
| Intelligence | ADHD | WM | -0.113 (0.026) | 0.89 [0.85-0.94] | 78 | NA | NA | NA |  | <0.001 |
| Intelligence | Anxiety disorder | MR-Egger | -0.153 (0.148) | 0.86 [0.64-1.15] | 76 | 0.532 | -0.036 | 0.004 | 0.316 | 0.306 |
| Intelligence | Anxiety disorder | WM | -0.021 (0.036) | 0.98 [0.91-1.05] | 76 | NA | NA | NA |  | 0.555 |
| Intelligence | Anxiety disorder | IVW | -0.005 (0.026) | 0.99 [0.95-1.05] | 76 | 0.531 | -0.022 | NA |  | 0.833 |
| Income | ADHD | MR Egger | 0.012 (0.175) | 1.01 [0.72-1.43] | 86 | <0.001 | 0.645 | -0.005 | 0.091 | 0.944 |
| Income | ADHD | IVW | -0.280 (0.035) | 0.76 [0.70-0.81] | 86 | <0.001 | 0.657 | NA |  | <0.001 |
| Income | ADHD | WM | -0.229 (0.036) | 0.80 [0.74-0.85] | 86 | NA | NA | NA |  | <0.001 |
| Income | Anxiety disorder | WM | -0.103 (0.047) | 0.90 [0.82-0.99] | 87 | NA | NA | NA |  | 0.028 |
| Income | Anxiety disorder | MR Egger | 0.020 (0.162) | 1.02 [0.74-1.40] | 87 | 0.931 | -0.292 | -0.003 | 0.375 | 0.901 |
| Income | Anxiety disorder | IVW | -0.121 (0.033) | 0.89 [0.83-0.95] | 87 | 0.932 | -0.277 | NA |  | <0.001 |
| EA | ADHD | IVW | -0.333 (0.018) | 0.72 [0.69-0.74] | 457 | <0.001 | 0.517 | NA |  | <0.001 |
| EA | ADHD | WM | -0.300 (0.020) | 0.74 [0.71-0.77] | 457 | NA | NA | NA |  | <0.001 |
| EA | ADHD | MR Egger | -0.249 (0.070) | 0.78 [0.68-0.89] | 457 | <0.001 | 0.515 | -0.001 | 0.217 | <0.001 |
| EA | Anxiety disorder | IVW | -0.056 (0.020) | 0.95 [0.91-0.98] | 454 | 0.425 | 0.011 | NA |  | 0.005 |
| EA | Anxiety disorder | WM | -0.070 (0.030) | 0.93 [0.88-0.99] | 454 | NA | NA | NA |  | 0.021 |
| EA | Anxiety disorder | MR Egger | -0.084 (0.085) | 0.92 [0.78-1.09] | 454 | 0.413 | 0.011 | 0 | 0.743 | 0.326 |
